# Proximity of Cytomegalovirus-Specific CD8^+^ T Cells to Replicative Senescence in Human Immunodeficiency Virus-Infected Individuals

**DOI:** 10.3389/fimmu.2018.00201

**Published:** 2018-02-15

**Authors:** John Joseph Heath, Neva Jennifer Fudge, Maureen Elizabeth Gallant, Michael David Grant

**Affiliations:** ^1^Division of BioMedical Sciences, Faculty of Medicine, Memorial University of Newfoundland, St. John’s, NL, Canada

**Keywords:** cytomegalovirus, human immunodeficiency virus, CD8^+^ T cell, senescence, telomere length, inflammation

## Abstract

Antiretroviral therapy (ART) effectively extends the life expectancy of human immunodeficiency virus (HIV)-infected individuals; however, age-related morbidities have emerged as major clinical concerns. In this context, coinfection with cytomegalovirus (CMV) accelerates immune senescence and elevates risk for other age-related morbidities, possibly through increased inflammation. We investigated potential relationships between CMV memory inflation, immune senescence, and inflammation by measuring markers of inflammation and telomere lengths of different lymphocyte subsets in HIV-infected individuals seropositive for anti-CMV antibodies. Our study cohort consists mainly of middle aged men who have sex with men (MSM) and heterosexuals who are stable under long-term ART. Median levels of IL-6, TNF-α, and CRP were significantly higher in those coinfected with CMV. Lymphocyte telomere length in general correlated with age, but for 32/32 subjects tested, there was a consistent hierarchy of telomere lengths with CD8^+^ T cells’ shorter than the general lymphocyte population, CD57^+^CD8^+^ T cells’ shorter than CD8^+^ T cells’ and CMV-specific CD57^+^CD8^+^ T cells’ the shortest of all. Telomeres of HIV-specific CD8^+^ T cells were longer than those of CMV-specific CD8^+^ T cells in all cases tested and over 10 years, CMV-specific CD8^+^ T cell telomeres of two HIV-infected individuals eroded faster than those of HIV-specific CD8^+^ T cells. These data indicate that CMV-specific CD8^+^ T cells of HIV-infected individuals are the lymphocytes closest to telomere-imposed replicative senescence. Exhaustive proliferation of CMV-specific CD8^+^ T cells in HIV-infected individuals is a potential source of senescent lymphocytes affecting systemic immune function and inflammation.

## Introduction

Infection with cytomegalovirus (CMV) is very common and usually problematic only in cases of congenital infection, immune suppression, or immune deficiency (1–5). However, following primary infection, human (H)CMV persists for life in latently infected cells with periodic reactivation contained by the immune system. Immune responses against CMV are generally robust and often undergo a process termed memory inflation, in which their magnitude increases disproportionately with age, compared to responses against other persistent viruses (6–9). In some old elderly individuals (>80 years), HCMV memory inflation produces an “immune risk profile” (IRP), characterized by pronounced accumulation of terminally differentiated HCMV-specific CD8^+^ T cells (10). The IRP manifests a CD4^+^/CD8^+^ T cell ratio below 1.0 and is accompanied by phenotypic and functional signs of immune senescence that signify elevated risk for all-cause morbidity and mortality (11–13). Broader population-based studies also associate HCMV infection with development of age-related morbidities, especially cardiovascular disease (14, 15). These findings raise the possibility that either through pro-inflammatory effects of viral reactivation or through long-term influence on immune system character, persistent HCMV infection promotes development of age-related morbidities.

Human immunodeficiency virus (HIV) infection, even when effectively controlled by combination antiretroviral therapy, accelerates HCMV immune memory inflation. This is reflected in earlier, more pronounced expansion of HCMV-specific CD8^+^ T cells and in greater NKG2C^+^CD57^+^ natural killer (NK) cell accumulation (16–18). Given the relationship between CMV immunity and all-cause mortality in the old elderly, the question arises as to whether the more pronounced immunological influence of HCMV in HIV infection relates to the more frequent and earlier onset of age-related morbidities in HIV infection (19–25). Recent studies indicate that HCMV coinfection is associated with more inflammation, less immune reconstitution, a history of greater CD8^+^ T cell proliferation, increased vascular intimal-medial thickening, and higher incidence of severe, non-AIDS neurovascular events in HIV infection (17, 26–32). However, any role for CMV-specific immunity as either marker or determinant of this association has not been directly examined.

We previously measured the frequency of T cell receptor excision circles (TREC) in peripheral blood T cell subsets of HIV-infected individuals and observed a lower median TREC frequency in CD8^+^ T cells of those coinfected with HCMV (32). There was also a significant inverse correlation between the size of CMV-specific CD8^+^ T cell responses and TREC frequency, suggesting a link between the exaggerated immune response against CMV and exhaustive T cell proliferation in HIV infection (32). In this follow-up study, we investigated the possibility of a direct link between CMV-specific immunity, CD8^+^ T cell proliferation, lymphocyte senescence, and inflammation. We first measured and compared plasma markers of inflammation in CMV-seropositive and seronegative HIV-infected individuals to assess any association between CMV seropositivity and apparently sterile systemic inflammation. We then selectively examined the telomeres of CMV-specific CD8^+^ T cells and other lymphocytes from a subset of these individuals for progress toward replicative senescence and potential acquisition of a senescence-associated secretory phenotype (SASP) (33). Our results affirm that CMV infection is associated with inflammation in HIV-infected individuals and position CMV-specific T cells at the leading edge of lymphocyte progression toward exhaustive proliferation and cellular senescence.

## Materials and Methods

### Study Subjects and Sample Collection

Subjects infected with HIV were recruited through the Newfoundland and Labrador Provincial HIV Clinic. All but several were MSM or heterosexual caucasians of western European descent with very few intravenous drug users. All subjects provided informed consent for whole blood collection, immunological studies, and researcher access to medical laboratory records. The Newfoundland and Labrador Health Research Ethics Authority approved this study. Routine clinical assessment with lymphocyte subsets including CD4^+^ and CD8^+^ T cell counts and viral load was performed at least once every 6 months. Prior to each clinic visit, whole blood was collected by forearm venipuncture into vacutainer tubes containing acid-citrate-dextrose anticoagulant. Plasma was collected following room temperature centrifugation of whole blood for 10 min at 400 × *g*, aliquoted and stored at −80°C until testing. Packed cells were diluted to two times the original blood volume with phosphate-buffered saline (PBS) and then peripheral blood mononuclear cells (PBMC) were isolated by Ficoll-Hypaque (GE Healthcare Bio-Sciences, Piscataway, NJ, USA) density gradient centrifugation, washed once in PBS with 1% fetal calf serum (FCS), and resuspended in lymphocyte medium comprising RPMI 1640 with 10% FCS, 100 IU/mL penicillin, 100 µg/mL streptomycin, 2 mM l-glutamine, 10 mM HEPES buffer solution, and 2.0 × 10^−5^ M 2-mercaptoethanol (all from Invitrogen, Carlsbad, CA, USA). Freshly isolated PBMC were resuspended at a minimum of 1.0 × 10^7^ cells/mL in freezing medium composed of lymphocyte medium with 10% dimethyl sulfoxide supplemented to 20% FCS and cooled at 1°C/min overnight to −80°C. Frozen PBMC were then maintained in liquid nitrogen until analysis. To thaw cells, cryopreserved PBMC were immediately immersed in 37°C water bath, gently agitated until almost thawed, then immediately transferred into, and washed three times in, 10 mL lymphocyte medium. Cells were then resuspended at 2.0 × 10^6^ cells/mL in lymphocyte medium and allowed to recover overnight at 37°C, 5% CO_2_. Cells were counted after recovery and used only when >70% were viable by trypan blue exclusion.

### Measurement of Anti-HCMV Antibodies

Subjects were separated into CMV-seropositive and CMV-seronegative groups based on the presence or absence of CMV-specific antibodies in plasma. The presence of CMV-specific IgG antibodies was assessed as previously described (18). Briefly, antibodies against HCMV were measured in plasma samples by ELISA against CMV AD169-infected MRC-5 cell lysate. To generate lysate, 1 × 10^7^ MRC-5 cells were infected with CMV AD169 at a multiplicity of infection of 0.5 and after 3 days were harvested by scraping, pelleted by centrifugation, and lysed in 1 mL lysis buffer. Lysate diluted 1/1,000 in carbonate/bicarbonate coating buffer was added in 100 µL overnight at 4°C to wells of Immunlon-2 ELISA plates (VWR Scientific, Mississauga, ON, Canada). Lysate prepared as above from uninfected MRC-5 cells was used as control. Plasma samples diluted 1/500 were incubated on the plates for 90 min, washed and developed with goat-anti-human IgG-horseradish peroxidase conjugate (Jackson ImmunoResearch Labs, West Grove, PA, USA) followed by tetramethylbenzidine substrate (Sigma-Aldrich). Color development ran for 30 min at room temperature, after which the reaction was stopped with 1 N H_2_SO_4_ and optical density read at 450 nm. CMV AD169 was obtained through the NIH AIDS Reagent Program, AIDS Program, NIAID, NIH from Dr Karen Biron (34). MRC-5 cells were a kind gift of Dr. K. Hirasawa, Division of BioMedical Sciences, Faculty of Medicine, Memorial University of Newfoundland.

### Measurement of Inflammatory Markers in Plasma

Fresh plasma was aliquoted to avoid repeat freeze/thaw cycles. Commercial ELISA kits were used to measure the following analytes in plasma as per the manufacturers’ instructions. Kits for measuring interleukin (IL)-1β (range = 2.00–200.00 pg/mL), IL-6 (range = 2.00–200.00 pg/mL), and tumor necrosis factor (TNF)-α (range = 4.00–500.00 pg/mL) were from eBioscience, San Diego, CA, USA. Kits for measuring C-reactive protein CRP (range = 15.60–1,000.00 pg/mL) were from R&D Systems, Minneapolis, MN, USA. The sensitivity ranges covered physiologically appropriate levels such that plasma was added neat to the assay, except for CRP where plasma was diluted 1:10,000 with PBS. All measurements were carried out in duplicate with control wells containing only PBS included in each assay. The OD value in control wells was subtracted from all test values to adjust for background. Absorbance was measured at 450 nm on a Biotek synergy HT ELISA reader and standard curves generated as specified.

### Peptide Stimulation of PBMC and Surface Marker Staining

All study participants had previously been tested for CD8^+^ T cell responses against CMV pp65 and immediate early-1 (IE-1) proteins separately using overlapping peptide sets (Peptivator, Miltenyi Biotec, San Diego, CA, USA) (18, 32). Most had also been tested for responses against individual HIV proteins with overlapping peptide sets (NIH AIDS Reagent Program, Germantown, MD, USA). Subjects with sufficiently strong responses for visualization by intracellular flow cytometry were selected to identify CMV-specific or HIV-specific CD8^+^ T cells for telomere measurement. If responses against both CMV IE-1 and pp65 were present, then PBMC were stimulated with the protein pools in aggregate and responses against the two proteins were not distinguished at this point. Aliquots of 2 × 10^6^ PBMC in 1 mL lymphocyte medium were stimulated with pooled CMV-pp65 (0.5 µg/mL) and IE-1 (0.5 µg/mL) peptide sets or overlapping HIV-1 Nef (1.0 µg/mL) and Gag (1.0 µg/mL) peptides (NIH AIDS Reagent Program, Germantown, MD, USA), for 60 min at 37°C (5% CO_2_). Brefeldin A (Sigma-Aldrich, St. Louis, MO, USA), was then added for a final concentration of 10.0 µg/mL and the PBMC left for an additional 4 h before being stained for surface markers and intracellular interferon-gamma (IFN-γ). Cells were washed twice in flow cytometry buffer consisting of PBS with 0.2% NaN3, 5 mM EDTA (Sigma-Aldrich), and 0.5% FCS, then incubated with fluorescein isothiocyanate-conjugated anti-human CD57 (TBO3, Miltenyi Biotec) and Quantum Dot 705-conjugated anti-human CD8 (3B5; Invitrogen) for 20 min at 4°C. Samples were kept in the dark from this point on. After another wash with flow buffer, cells were fixed and permeabilized with InsideStain (Miltenyi Biotec) according to manufacturer’s instructions and intracellular staining with allophycocyanin-conjugated anti-human IFN-γ (4S.83, eBioscience) was carried out.

### PBMC Subset Telomere Length Measurement by Flow Cytometry

Measurement of telomere length by flow cytometry was carried out with minor adaptation of a previously described protocol (35). Following surface and intracellular staining, samples were resuspended in 250 µL 1% FCS-PBS on ice. To increase the stability of antigen–antibody–conjugate complexes, 7.5 mM bissulfosuccinimidyl suberate (BS_3_) crosslinking solution (ThermoFisher Scientific, Waltham, MA, USA) in PBS was added to a final concentration of 5 mM and incubated on ice for 30 min. Residual BS3 was quenched with 3 mL quenching solution (100 mM Tris–HCl, 50 mM NaCl in PBS). Samples were incubated on ice for a further 20 min and standard 1301 T leukemia cells (Sigma-Aldrich) were added at a ratio of 1:4 to sample PBMC. Samples were then washed in flow buffer, split into two aliquots, and transferred to fresh 1.5 mL microcentrifuge tubes, for a probed and unprobed control sample. Samples were pelleted and all but 100 µL supernatant removed *via* pipette to ensure pellet stability. Then, 250 µL of hybridization solution (70% formamide, 30 mM Tris–HCl, 0.2 M NaCl, 1.5% BSA) was added and samples were resuspended with a wide bore 1 mL pipettor and incubated for 10 min at RT. All subsequent resuspensions were done in this manner to avoid unnecessary shear force on fragile samples. Samples were centrifuged at 1,600 × *g* to ensure optimal pellet formation in formamide without compromising cellular integrity. All but 100 µL of supernatant was again removed *via* pipette. Samples were then resuspended in 250 µL hybridization solution with or without the addition of 0.75 µg/mL TelC-Cy3 (AATCCC)_3_ (Panagene Inc., Daejeon, Korea). An unprobed control to allow correction for formamide-related auto-fluorescence that may artificially increase Cy3 fluorescence was run with every sample. All aqueous reagents were verified pH 7.2 and sterile filtered through a 0.45 µm nylon filter prior to formamide addition.

Samples were then incubated at 84°C for 10 min, placed on ice for 5 min and left to hybridize in a dark chamber for 2 h at RT. Samples were then diluted 3:1 with a post-hybridization solution (70% formamide, 15 mM Tris–HCl, 0.2 M NaCl, 0.15% BSA, 0.15% Tween-20) and centrifuged at 1,600 × *g*. All but 100 µL of supernatant was removed *via* pipette and samples were washed twice with 1% BSA, 0.5 mM EDTA in PBS, centrifuging first at 900 × *g* and then at 500 × *g* to ensure maximal removal of formamide. Samples are then resuspended in the same wash solution and analyzed immediately with a FACSCalibur Cell Analyzer (BD Biosciences, San Jose, CA, USA). A minimum of 1 × 10^5^ events were acquired per sample.

### Calculation of Telomere Length

Although the standard cells were always run together with test samples, representing an internal standard in each telomere length assay, a probed and unprobed sample of 1301 standard cells was also run separately to calculate intra-assay variation in the mean fluorescence intensity (MFI) measured for the standard cells. The SD in geometric MFI for the 1301 cells across 32 assays was 16%. Using Cy3 MFI of the 1301 control cell line and of the sample subsets, the relative and absolute telomere length of the subset is calculated using the known 1301 telomere length [23,480 base pairs (bp)] and the following formula.

$$\begin{array}{l} \text{Relative Telomere Length }(\text{RTL})\\ =\frac{\left(({\text{MFI}}_{\text{Cy}3}({\text{Sample}}_{\text{probe}})-{\text{MFI}}_{\text{Cy}3}({\text{Sample}}_{\text{unprobed}}))\times \text{DNA Index of }1301\right)}{\left(({\text{MFI}}_{\text{Cy}3}(1301_{\text{probe}})-{\text{MFI}}_{\text{Cy}3}(1301_{\text{unprobed}}))\times {\text{DNA Index of }}_{\text{Sample}}\right)}=\frac{{\text{SampleTL}}_{\text{MFI}}}{1301{\text{TL}}_{\text{MFI}}} \end{array}$$

$$\text{Exact Telomere Length}(\text{TL})\text{=}\frac{{\text{SampleTL}}_{\text{MFI}}}{{\text{1301TL}}_{\text{MFI}}}\times {\text{1301TL}}_{\text{bp}}$$

### Statistical Analysis

Statistical analyses were carried out using Prism version 6 (GraphPad Software, Inc., La Jolla, CA, USA). Normal distribution of data was assessed by the Shapiro–Wilk test. If deviation from normal distribution was indicated (all TL results, IL-1β, IL-6, TNF-α), data were represented with median ± interquartile range (IQR) and group medians compared by Mann–Whitney test or Wilcoxon-signed rank test. Sex distribution in CMV-seropositive compared to CMV-seronegative groups was compared by Fisher’s exact test. Spearman correlation was used to assess relationships between variables. If data were normally distributed (age, CD8^+^ T cell response), mean ± SD was calculated and Student’s *t*-test was used to compare means. Relationships were assessed using Spearman correlation matrices.

## Results

### Markers of Inflammation in HIV-Infected Groups Distinguished by CMV Infection Status

**Figure 1:**
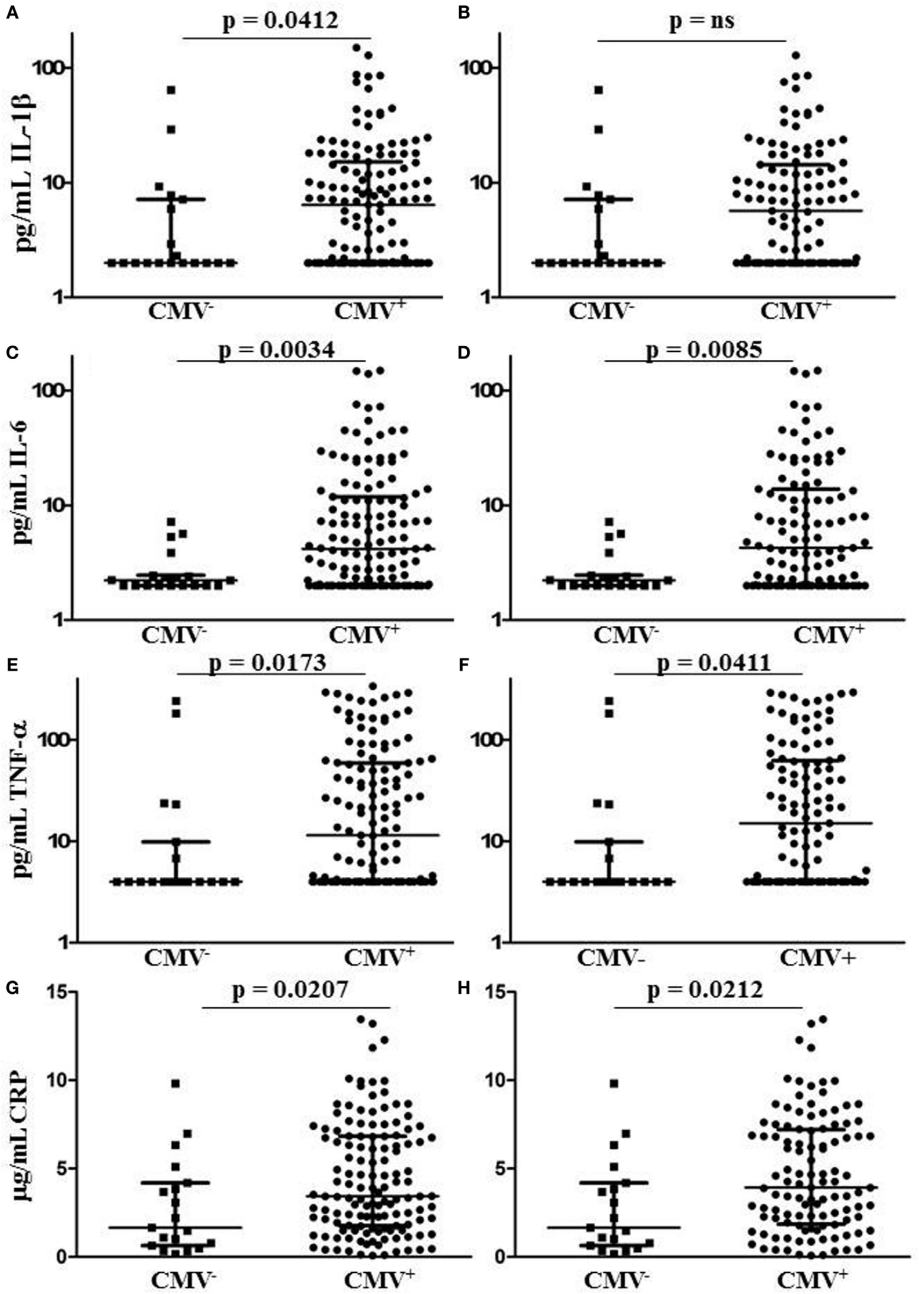
Plasma levels of CRP and pro-inflammatory cytokines IL-1, IL-6, and tumor necrosis factor (TNF)-α in human immunodeficiency virus (HIV)-infected individuals grouped by cytomegalovirus (CMV) seropositivity status. Plots **(A,C,E,G)** show results for all HIV-infected individuals tested, while plots **(B,D,F,H)** show only those individuals with no detectable HIV plasma virus load within the 12 months immediately preceding testing. Horizontal lines bisecting the groups show median values for each measure with interquartile range shown above and below. Significant differences between medians (Mann–Whitney test) are shown above lines spanning the groups compared.

After excluding individuals coinfected with hepatitis C virus (HCV), we measured levels of IL-1, IL-6, TNF-α, and CRP in plasma samples from 153 HIV-infected individuals characterized for CMV infection status, age, gender, duration of infection, antiretroviral therapy (ART), lymphocyte subset counts, plasma HIV load, CMV-specific T cell immunity, and T cell subset TREC frequency. Of the 153 HIV-infected subjects included, 134 were seropositive for anti-CMV antibodies and 20 of these 134 had at least 1 detectable HIV plasma virus load in the 12 months immediately preceding testing. Three of the 19 individuals seronegative for CMV had at least 1 detectable HIV plasma virus load within the 12 months immediately preceding testing. General demographic characteristics of the subjects with comparisons between the CMV-seropositive and seronegative groups are shown in Table 1. Detectable HIV replication was considered any level above the detection limit of the commercial assays used, usually 50 copies HIV RNA/mL plasma, at any one time point within 12 months of the plasma collection for inflammatory marker measurement. Whether individuals with detectable HIV replication within the last 12 months of clinical plasma viral load testing were excluded or not, median IL-6, TNF-α, and CRP levels were significantly higher in the subset coinfected with CMV (Figures 1C–H). Median levels of IL-1β were only significantly higher in the CMV-infected group when those individuals with detectable HIV replication within the last 12 months were included in the analysis (Figures 1A,B). There were significant correlations between IL-6 levels and age (*r* = 0.484, *p* = 0.007) and between CRP levels and age (*r* = 0.403, *p* = 0.027), but there was no significant difference between median ages of the CMV-seropositive [48 years, interquartile range (IQR) 44–54] and seronegative groups (44 years, IQR 42–50). These data show a broad range in levels of common markers of inflammation in HIV-infected individuals with increased median levels of pro-inflammatory cytokines IL-6 and TNF-α and of the acute-phase protein CRP in the group coinfected with CMV. These differences in inflammatory markers were independent of detectable HIV replication within the 12 months immediately preceding testing. In our HIV-infected study cohort, coinfection with CMV is associated with greater inflammation, unrelated to clinically apparent effects on HIV replication.

**Table 1:** General characteristics of study cohort and comparison of cytomegalovirus (CMV)-infected and uninfected.

	CMV-seronegative		CMV-seropositive		*p*
*n* (% undetectable human immunodeficiency virus at time of testing)	19	(84%)	134	(77%)	0.5459
*n* (% male)	11	(61%)	103	(76%)	
*n* (% female)	8	(39%)	31	(24%)	0.0929
Age (years), median interquartile range (IQR)	44	(42–50)	48	(44–54)	0.1165
β-2 microglobulin (μg/mL), median (IQR)	2.63	(2.06–3.24)	2.64	(2.08–3.33)	0.9082
% CMV-specific CD8^+^ T cells, mean (SD)	0.01	(0.03)	3.94	(4.15)	<0.0001
Duration of antiretroviral therapy (years), median (IQR)	13	(8–19)	15	(10–19)	0.3753
Nadir CD4^+^ T cells/μL blood, median (IQR)	190	(86–325)	234	(121–404)	0.3655
CD4^+^ T cells/μL blood, median (IQR)	742	(522–780)	648	(419–777)	0.7689
CD8^+^ T cells/μL blood, median (IQR)	648	(442–770)	869	(643–1,217)	0.0093
CD4^+^:CD8^+^ T cell ratio, median (IQR)	1.05	(0.86–1.64)	0.68	(0.44–0.92)	0.1273

### Lymphocyte Subset Telomere Lengths in HIV-Infected Individuals

**Figure 2:**
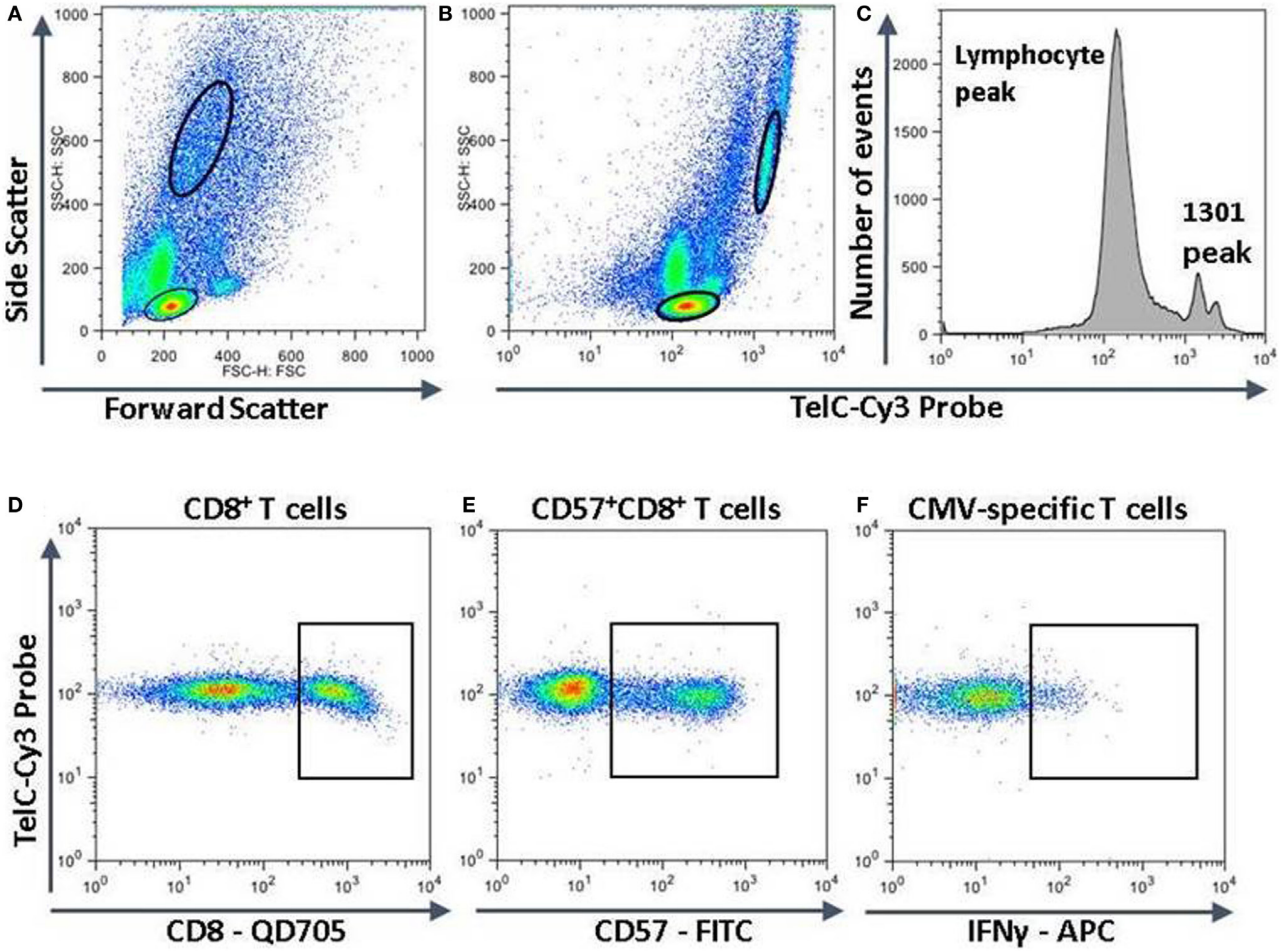
Measurement of telomere length in different lymphocyte subsets. Peripheral blood mononuclear cells were surface stained with fluorescent labeled antibodies against CD8 and CD57, then together with 1301 standard cells were fixed, permeabilized, and subjected to hybridization with a fluorescent Cy3-labeled telomere-specific DNA probe. If stimulated with human immunodeficiency virus or cytomegalovirus (CMV) peptides, they were stained for intracellular interferon-gamma (IFN-γ) before hybridization. The strategy for subset analysis was as shown with successive gating to identify lymphocytes and 1301 cells **(A,B)**, CD8^+^ T cells **(D)**, CD57^+^CD8^+^ T cells **(E)**, and CMV-specific CD57^+^CD8^+^ T cells producing IFN-γ **(F)**. **(C)** The fluorescence intensities of the entire lymphocyte population and 1301 standard cells. The major 1301 peak represents cells in the G1 phase of the cell cycle while the smaller peak to the right represents cells in S or later phases of the cell cycle.

**Figure 3:**
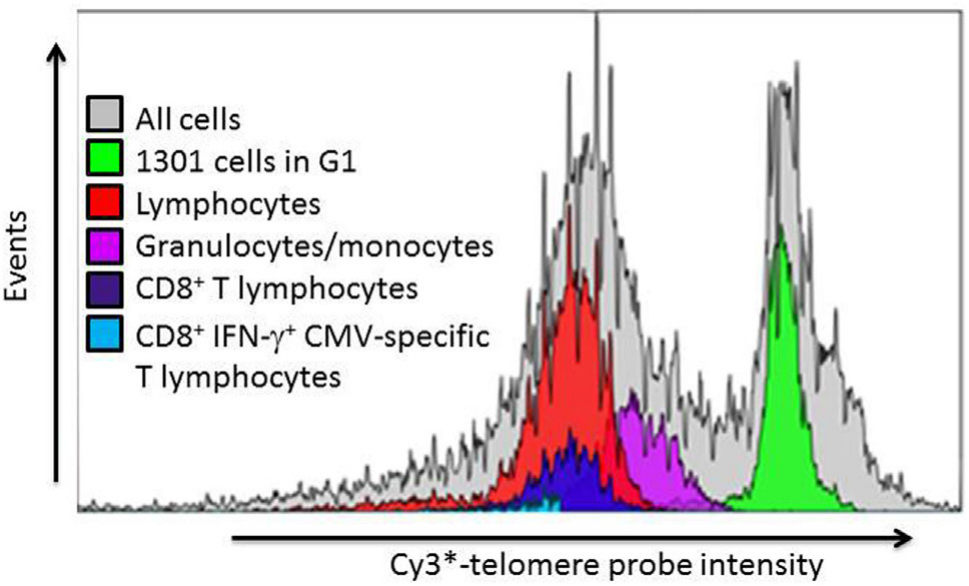
Representative example of leukocyte subset telomere probe fluorescence intensity distribution. After gating on lymphocyte subsets as indicated in Figure 3, the telomere probe fluorescence intensity distribution of each individual subset is expressed as a histogram from which the geometric mean fluorescence intensity (MFI) is derived. The telomere length of each subset is then estimated as described in Section “Materials and Methods” from the ratio of subset MFI to that of the internal standard 1301 cells in G1 phase of the cell cycle.

**Figure 4:**
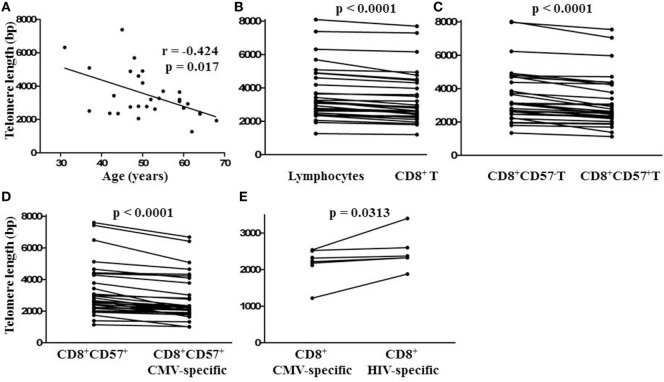
Relationship between lymphocyte telomere length and age and comparison of lymphocyte subset telomere lengths within individuals. **(A)** Spearman non-parametric correlation indicated significant negative correlation between lymphocyte telomere length and age. The correlation coefficient (*r*) and probability of significant correlation (*p*) are shown in the plot frame. Linear regression was done to generate the line of best fit. **(B)** Points representing median telomere lengths for lymphocyte and CD8^+^ T cell subsets, **(C)** CD8^+^ and CD8^+^ CD57^+^ T cell subsets, **(D)** CD8^+^CD57^+^ and CD8^+^CD57^+^ cytomegalovirus (CMV)-specific T cell subsets, and **(E)** CD8^+^ CMV and human immunodeficiency virus (HIV)-specific T cell subsets joined by horizontal lines showing differences between subsets within each individual. Significant differences between subset telomere lengths are shown above the plot frames (Wilcoxon-signed rank test).

To compare the impact of proliferative history on different lymphocyte subsets in relation to CMV immunity and inflammation, we measured telomere length by fluorescence *in situ* hybridization (FISH) flow cytometry in lymphocytes of a representative set of HIV-infected individuals seropositive for CMV-specific antibodies (Table 2). These individuals had cellular immune responses against CMV ranging from 0.2 to 32% of their CD8^+^ T cells. Sequentially more exclusive analysis gates were applied starting with lymphocytes and proceeding through CD8^+^ lymphocytes, CD8^+^CD57^+^ lymphocytes, and antigen-specific CD8^+^CD57^+^ lymphocytes identified by intracellular IFN-γ production (Figure 2). Telomere length in nucleotide bp was estimated by the ratio of subset telomere probe geometric MFI to the geometric MFI of the telomere probe hybridized to the 1301 standard control (27.5 kb telomeres) cell line analyzed concurrently (Figure 3). There was a broad range of telomere lengths in subjects tested with a significant correlation between lymphocyte telomere length and age (*r* = −0.424, *p* = 0.017, Figure 4A). The CD8^+^ T cells had shorter telomeres than the rest of the lymphocyte population (*p* = 0.0018, Figure 4B) and CD57^+^CD8^+^ T cells had shorter telomeres than CD8^+^CD57^-^ T cells (*p* < 0.0001, Figure 4C). These data affirm results of previous lymphocyte subset telomere length assessment studies in HIV infection and validate our application of the FISH flow cytometry assay for telomere length in this setting (36–38).

**Table 2:** General characteristics of cytomegalovirus (CMV)-seropositive subjects selected for telomere length analysis.

*n* (% undetectable human immunodeficiency virus at time of testing)	32	(74)
*n* (% male)	24	(75)
*n* (% female)	8	(25)
Age (years), median [interquartile range (IQR)]	50	(47–59)
% CMV-specific CD8^+^ T cells, mean (±SD)	4.35	4.00
Duration of antiretroviral therapy (years), median (IQR)	15	(8–20)
Nadir CD4^+^ T cells/μL blood, median (IQR)	218	(60–304)
CD4^+^ T cells/μL blood, median (IQR)	651	(492–763)
CD8^+^ T cells/μL blood, median (IQR)	888	(620–1,226)
CD4^+^:CD8^+^ T cell ratio, median (IQR)	0.67	(0.45–0.96)

### Telomere Lengths of CMV-Specific CD8^+^ T Cells

To position CMV-specific CD8^+^ T cells along the lymphocyte telomere length continuum for each individual and address the potential relationship between CMV-specific immunity and exhaustive CD8^+^ T cell proliferation, we used CMV peptides to stimulate PBMC from HIV-infected individuals seropositive for CMV prior to FISH. We then compared telomere lengths of CMV-specific CD8^+^CD57^+^ T cells producing IFN-γ and the remaining CD8^+^CD57^+^ T cells. In 32/32 cases tested, the telomeres of CMV-specific CD8^+^CD57^+^ T cells were shorter, by several hundred to several thousand bp (Figure 4D, *p* < 0.0001). To compare telomere lengths of CMV-specific T cells with another antigen-specific T cell subset, we stimulated the PBMC of a group of individuals with strong HIV-specific CD8^+^ T cell responses with CMV peptides or appropriate HIV peptides prior to FISH and compared telomere lengths of HIV-specific and CMV-specific CD8^+^ T cells. In all cases studied, CMV-specific T cells had shorter telomeres than HIV-specific T cells of the same individual (*p* < 0.05, Figure 4E). These data indicate by telomere length analysis that CMV-specific T cells of HIV-infected individuals are the T cells most proximal to exhaustive proliferation and replicative senescence.

**Figure 5:**
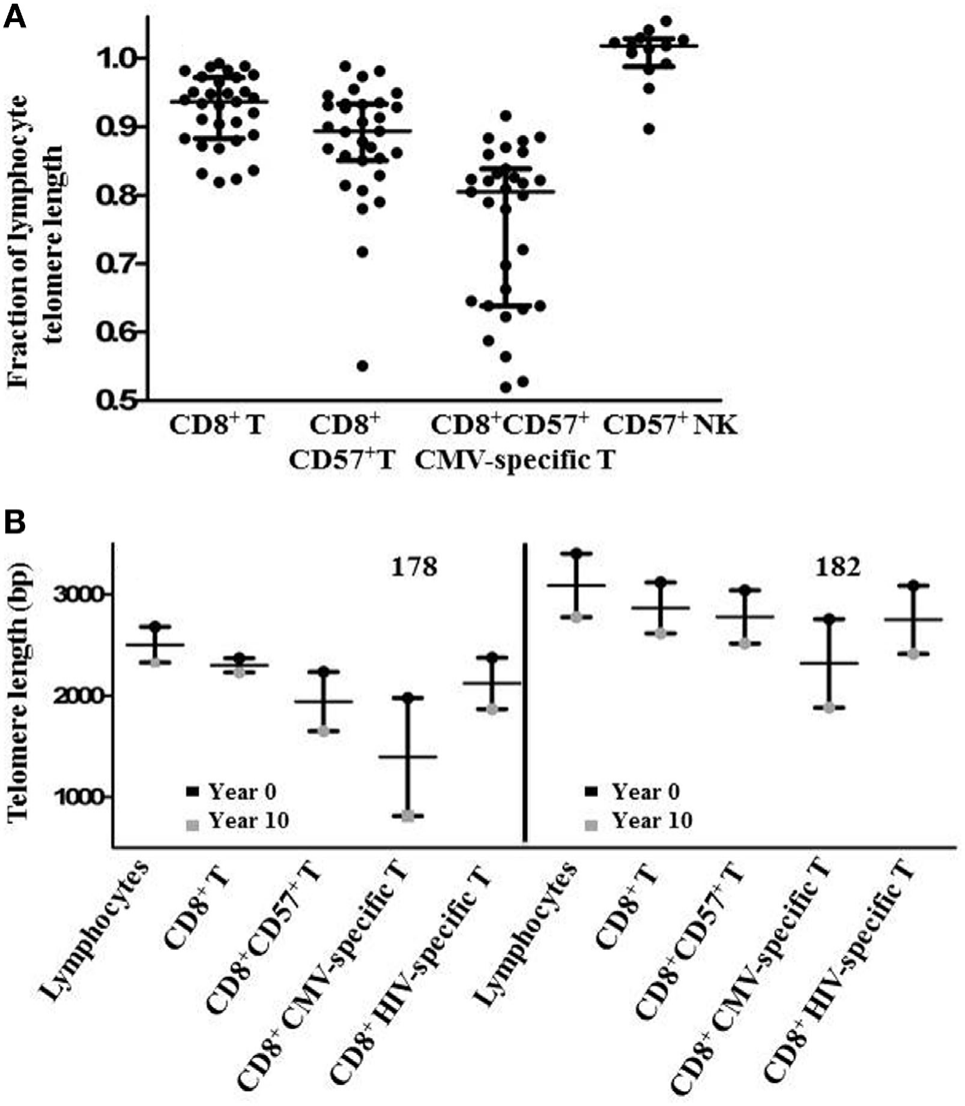
Subset telomere length relative to the lymphocyte population. **(A)** Lymphocyte subset median telomere lengths are shown as a fraction of the total lymphocyte population median telomere length for each individual to normalize values across different ages and genetic backgrounds. Horizontal lines bisecting the groups show median values with interquartile range shown above and below. **(B)** Lymphocyte subset telomere length measurements for two subjects were carried out on peripheral blood mononuclear cells samples collected approximately 10 years apart. Vertical lines represent the loss of telomere length for each subset.

Since there was a broad range of absolute telomere lengths related to subjects’ ages and genetic variation, for broader comparison, we normalized the telomere length of different lymphocyte subsets by expressing it as a fraction of the median length of the general lymphocyte population telomeres for each individual. This representation clearly shows successive relative shortening of telomeres through CD8^+^, CD8^+^CD57^+^, and CD8^+^CD57^+^ CMV-specific T lymphocyte subsets (Figure 5A). It also clearly illustrates relative preservation of telomere length within the CD8^+^ T cell subset and within the CD57^+^ NK subset compared to CD8^+^CD57^+^ T cells and CMV-specific CD8^+^CD57^+^ T cells (Figure 5A). Longitudinal sampling of telomere lengths over 10 years within CMV and HIV-specific CD8^+^ T cells of two individuals for whom cryopreserved PBMC were available (subjects 178 and 182) showed accelerated erosion of CMV-specific CD8^+^ T cell telomeres compared to the overall lymphocyte population, CD8^+^ T cells, CD8^+^CD57^+^ T cells, and HIV-specific CD8^+^ T cells (Figure 5B). Both of these individuals were male caucasians infected with HIV for >20 years. For subject 182, the period of telomere measurement spanned age 42–51. He has been receiving ART for 16 years with no recorded detectable HIV replication over the study period. For subject 178, the period of telomere measurement spanned age 59–68. He has been receiving ART for 13 years with two recorded minor transient blips of HIV replication over the study period.

To investigate a direct relationship between CMV immunity-related telomere erosion and inflammation, we assessed correlation between the relative telomere lengths (fraction of the median length of the general lymphocyte population) of different lymphocyte subsets and plasma levels of pro-inflammatory cytokines and CRP. We observed a significant inverse correlation between relative telomere length of CMV-specific CD57^+^CD8^+^ T cells and plasma levels of CRP (*r* = −0.4756, *p* = 0.0122). This suggests that telomere erosion of CMV-specific CD8^+^ T cells in HIV-infected individuals is related to systemic inflammation.

## Discussion

Premature functional deterioration of multiple physiological systems, including the immune system, is a primary concern for the extended health of HIV-infected individuals (20, 22, 23, 39). Factors related to immune dysfunction and persistent inflammation may contribute to the earlier and more frequent incidence of age-related morbidities in the HIV-infected population (21, 40). Based on notable associations between CMV infection and cardiovascular disease in the general population and between CMV infection and immune senescence in old elderly, we and others have investigated the relationship between coinfection with CMV, systemic inflammation, and age-related morbidity in HIV infection (27, 28, 30–32, 41). In this study, we specifically investigated the relationship between CD8^+^ T cell immunity against CMV and lymphocyte progression toward replicative senescence imposed by telomere loss. If replicative senescence of lymphocytes introduces a SASP, similar to that of other senescent cells, this could promote persistent systemic inflammation and provide a mechanistic link between CMV infection and an increased risk for certain age-related morbidities. After excluding subjects infected with HCV, we compared plasma levels of CRP and of several pro-inflammatory cytokines between groups of HIV-infected infected individuals distinguished by their CMV seropositivity status. Since we did not test for CMV DNA or conduct more sensitive ELISPOT assays to detect T cell responses against CMV, it is possible that some of the 19 CMV-seronegative subjects were not completely CMV-negative. However, we feel this is unlikely based on the consistency with which seropositivity accompanied T cell responses detectable by flow cytometry. Although there was a broad range in the levels of these inflammatory markers, median IL-6, TNF-α, and CRP levels were all significantly higher in the group seropositive for CMV. Levels of IL-1 were significantly higher only when individuals with detectable HIV replication within 12 months of testing were included in the analysis. Thus, elevated IL-1 levels in the coinfected group may be associated with factors favoring HIV replication more so than with CMV coinfection itself. However, the elevated levels of IL-6, TNF-α, and CRP that were independent of detectable HIV replication support an association between CMV seropositivity and inflammation that could be relevant to the pathogenesis of accelerated aging and of particular age-related morbidities.

The relationship between CMV infection and immune senescence in the old elderly arises through a memory inflation process wherein accumulation of CMV-specific CD8^+^ T cells lowers the circulating CD4^+^/CD8^+^ T cell ratio below 1 (10, 11). Oligoclonal T cell proliferation underlies this accumulation; therefore, CD8^+^ CMV-specific T cells with the most extensive history of cell division may be approaching replicative senescence imposed by telomere erosion (7, 8). Previous research with non-HIV-infected individuals indicated that CMV-specific T cells did not suffer senescence imposed by telomere shortening (35). Accelerated CMV memory inflation in HIV infection coincides with increases in inflammation and in age-related morbidities, but no direct links have been investigated (16, 18). We speculated that acquisition of a SASP by CMV-specific, or other senescent T cells accumulating through extensive cell division, could link CMV memory inflation to systemic inflammation and its downstream sequelae. Callender et al. recently showed that senescent T cells could be identified by expression of a disintegrin and metalloprotease domain (ADAM) 28 and that they produce high levels of pro-inflammatory cytokines, especially TNF-α (42). In our study, telomere length analysis demonstrated that CD8^+^ CMV-specific T cells are the circulating lymphocytes nearest to telomere-imposed replicative senescence in HIV-infected individuals, but replicative senescence was not directly addressed. Senescent cells have been proposed as a source of the chronic low-grade inflammation related to immune senescence and development of age-related morbidity (43, 44). In this regard, we found significant correlations of relative telomere length of CD57^+^CD8^+^ CMV-specific T cells with age and with plasma levels of CRP, but no significant correlation with any of the other markers of inflammation we measured. There was also no significant correlation between relative telomere length of CD57^+^CD8^+^ CMV-specific T cells and either concurrent or nadir CD4^+^ T cell counts. We focused on CD8^+^ CMV-specific T cells in gauging perisenescence as previous research showed that CD8^+^ but not CD4^+^ T cells of HIV-infected individuals have shorter telomeres than those of their HIV-discordant twins (37). We also previously showed that the CD8^+^ but not CD4^+^ T cells of CMV-seropositive HIV-infected subjects have lower T cell receptor excision circle frequencies than those of age-matched CMV-seronegative HIV-infected subjects (32). Both findings indicate more extensive proliferation and closer proximity to replicative senescence of CD8^+^ T cells in HIV-infected individuals.

While this snapshot of peripheral blood lymphocyte telomere length could suggest a direct relationship between CMV-related exhaustive lymphocyte proliferation and systemic inflammation in HIV infection, other factors may complicate or obscure the relationship and also limit the power of our study to link immune senescence, inflammation, and age-related morbidity in HIV infection to CMV immunity. Senescent lymphocytes may undergo rapid clearance, may home from the bloodstream to different tissues such as the liver, or may simply not acquire a SASP that notably impacts levels of the pro-inflammatory cytokines we measured. Accumulation of perisenescent CMV-specific CD8^+^ T cells may itself have prognostic significance. Further characterization of CMV-specific CD8^+^ T cells for expression of the senescence-associated proteins p16Ink4a or *FOXO4a* may shed light on their status and allow for finer discrimination of any relationship to markers of systemic inflammation (45, 46). Other inherent limitations to our study involve the primarily cross-sectional nature of our study which does not specifically control for such aspects as previous levels of disease progression, duration of HIV infection, duration of CMV infection, duration of ART, history of CMV reactivation, age, sex, and lifestyle. Thus, these results may apply selectively to the particular HIV-infected population we studied. However, as comparisons of CMV-specific CD8^+^ T cell telomere length with that of other lymphocytes were always carried out within individuals, thus, serving as their own controls, and the results were consistent across 32/32 subjects, we are confident in the strength of our conclusion that the CMV-specific cells have undergone the most extensive proliferation and are most proximal to telomere-imposed senescence in this setting.

As CMV-specific T lymphocytes are the largest antigen-specific T cell subset and have the shortest telomeres of all lymphocytes, they represent a highly accessible contemporary record of chronic or recurrent immune activation events that have driven clonal proliferation. The extent of telomere attrition relative to that of HIV-specific T cells is somewhat surprising, given the almost universal chronicity of HIV replication in the absence of effective treatment. Overall, antigen-driven proliferation might be expected to be higher in the HIV-specific T cell subset, but CMV could have been acquired much earlier in life than HIV infection for most individuals and the T cell response may be more oligoclonal than that mounted against HIV. We have also speculated that CMV-specific T cells may have a selective advantage over other T cells in homeostatic T cell proliferation, driven by overall lymphocyte attrition and the cytokine environment established in chronic HIV infection. The rate of telomere attrition over 10 years was higher among CMV-specific CD8^+^ T cells than either HIV-specific CD8^+^ T cells or the general CD8^+^ T cell population in both individuals that we studied and neither had experienced any clinically apparent CMV reactivation. Both a higher rate of CMV reactivation and increased homeostatic T cell proliferation could contribute to the accelerated CMV memory inflation occurring in HIV-infected individuals.

While the shortened telomeres of CMV-specific CD8^+^ T cells directly reflect immunological history as it pertains to CMV, it will be important to determine whether lingering senescent or perisenescent cells portray or influence aspects of immune senescence beyond their own status. Accumulation of CMV-specific lymphocytes in old elderly signifies a global decline in immune function and reduced survival expectancy. It remains unclear whether the accumulation of CMV-specific lymphocytes is simply a symptom associated with generalized immune decline or is itself a driver of immune decline. While there is no direct evidence that CMV-specific lymphocytes contribute to systemic inflammation, their association with morbidity and mortality and positioning at the leading edge of lymphocyte progress toward cellular senescence is consistent with a negative influence on the function of other immune cells they interact with. We and others have already shown that immune resilience and immune reconstitution is superior in HIV-infected subjects seronegative for CMV (17, 28). The global decline in T cell receptor excision circle (TREC) frequency in CD8^+^ T cells is significantly correlated with magnitude of the CD8^+^ CMV-specific T cell response in HIV-infected individuals and higher anti-CMV antibody levels are associated with frailty (14, 32, 47). Although it remains only an association, stronger CMV-specific immune responses clearly have negative implications for the health of aging individuals.

Nonetheless, it is possible to alternatively interpret our findings as the increased inflammation in HIV infection due to bacterial product translocation from the gut or other causes promoting CMV replication, immune memory inflation, and progression of CMV-specific CD8^+^ T cells toward replicative senescence. To complicate matters further, a recent study demonstrated CMV replication in the intestinal epithelium of HIV-infected individuals, showed that intestinal epithelial cells are fully permissive to CMV infection and found that transepithelial permeability *in vitro* increased with CMV infection (48). Thus, it is also possible that CMV replication in the gut is itself a contributor to the loss of gastrointestinal barrier integrity that is linked with systemic inflammation in HIV-infected individuals (49). In this scenario, the accelerated CMV immune memory inflation seen in HIV infection would be a collateral effect of systemic inflammation, rather than a cause, as we propose.

In order to distinguish effects related to the progress of CMV-specific T cells toward senescence from other effects of aging, age-matched CMV-seronegative HIV-infected control subjects are a critical control group. Many other aspects of immune function beyond immune reconstitution remain to be compared between CMV-infected and CMV-seronegative groups. To investigate a direct role for CMV-specific T cells, the effect of their deletion on the function of other immune cells should be studied *in vitro* as several previous studies reported CMV-specific T cells with a T-regulatory cell phenotype (50–52). In addition, it could be informative to compare the replicative histories of CMV-specific T cells with differing fine specificity for pp65 or IE-1 peptides, rather than group all CMV IE-1 and pp65-specific CD8^+^ T cells together as we did. Identifying CMV-specific T cells at the leading edge of progress toward senescence also provides an opportunity to observe sequential changes in T lymphocytes as they reach replicative senescence and to define the SASP, if such exists, associated with senescent lymphocytes arising *in vivo* in a setting of chronic infection. Observations on immune senescence and its systemic impact made through the intensifying lens of HIV/CMV coinfection may be broadly applicable to survivors of chemotherapy, graft *versus* host disease, advanced aging, and other chronic conditions.

## Ethics Statement

All subjects gave written informed consent in accordance with the Declaration of Helsinki. The protocol was approved by the Health Research Ethics Authority of Newfoundland and Labrador.

## Author Contributions

MDG conceived the study and drafted the manuscript together with JH. JH performed the telomere length measurements, pro-inflammatory marker ELISAs, and some CMV-specific peptide stimulations. NF performed most of the CMV-specific peptide stimulations and all serologic testing for CMV-specific antibodies. NF and MEG processed and archived all samples for this study.

## Conflict of Interest Statement

The authors declare that the research was conducted in the absence of any commercial or financial relationships that could be construed as a potential conflict of interest.
